# 

**Published:** 1952-10

**Authors:** 


(CONTAINS INDEX)

### THE BRISTOL MEDICO-CHIRURGICAL JOURNAL

ARMED
FORCES
MAY 12 1953
MEDICAL
LIBRARY

*October 1952*

VOL. 69. No. 252 Price 4/-

